# A novel mechanism of HIV transcytosis and infection

**DOI:** 10.1186/1742-4690-9-S2-P206

**Published:** 2012-09-13

**Authors:** S Gupta, JC Becerra, DN Forthal

**Affiliations:** 1 University of California Irvine, Irvine, CA, USA

## Background

Female genital tract mucosae are bathed in acidic secretions, which, in the case of HIV-1 infected women, likely contain virus coated with antibody. Thus, uninfected male sexual partners are often exposed to an acidic milieu containing virus in the form of immune complexes. We investigated the impact of low pH and antibody on transcytosis, a potentially critical mechanism by which HIV-1 passes through genital tract epithelia to infect susceptible target cells.

## Methods

HIV-1 was incubated with Env-specific monoclonal and polyclonal antibodies at pH 6.0 or pH 7.4 and exposed to the apical surface of tight junction-forming human endometrial carcinoma (HEC-1) cells in transwell plates. The quantity and infectivity of transcytosed virus was measured by RT-PCR and infection of TZMbl cells, respectively.

## Results

We found that the combination of acidic pH and Env-specific antibody augmented transcytosis as much as 30-fold compared with Env-specific antibody at neutral pH or compared with non-specific antibody or no antibody at neutral or acidic pH. The pH and antibody dependence of enhanced transcytosis was blocked by antibody specific for the Fc neonatal receptor (FcRn) or by treatment with bafilomycin A1 (which inhibits acidification of endosomes). Non-neutralizing antibodies resulted in a lower quantity of total transcytosed virus, measured by RT-PCR, than did neutralizing antibodies. However, the ratio of total to infectious virus was much higher for neutralizing antibodies, indicating that neutralizing antibodies efficiently allow transcytosis while blocking infectivity of the transcytosed virus; the non-neutralizing antibodies facilitate transcytosis (although to a lesser degree than the neutralizing antibodies) without blocking infectivity.

## Conclusion

These results demonstrate that acidity and Env-specific antibody greatly enhance transcytosis of virus across mucosal epithelial cells via FcRn. Since male penile and urethral tissues express FcRn, our results suggest a novel mechanism by which antibody, and in particular, non-neutralizing antibody, might facilitate female-to-male transmission following sexual exposure.

